# Observation of Schlemm’s canal and transluminal trabeculotomy using an ophthalmic endoscope: a case report

**DOI:** 10.1186/s13256-019-2186-5

**Published:** 2019-08-11

**Authors:** Isao Nakao, Tadashi Mine, Mika Sakaguchi, Hiroshi Enaida

**Affiliations:** Department of Ophthalmology, Faculty of Medicine, Saga University, Saga, 849-8501 Japan

**Keywords:** Transluminal trabeculotomy, Ab interno trabeculotomy, Ophthalmic endoscopy, Corneal opacity, Cervical osteoarthritis

## Abstract

**Background:**

Gonioscopy-assisted transluminal trabeculectomy is a novel and useful technique for ab interno trabeculotomy. However, gonioscopy-assisted transluminal trabeculectomy is difficult to perform in patients with corneal opacity or in patients with sequelae of cerebral infarction and cervical osteoarthritis with severe limitation of spinal mobility. This is because observing Schlemm’s canal during surgery using gonioscopy is difficult. In this report, we introduce a new and beneficial surgical technique of transluminal trabeculotomy for these patients, using an ophthalmic endoscope for cases in which normal gonioscopy-assisted transluminal trabeculectomy is difficult.

**Case presentation:**

Our patient was a 65-year-old Japanese man with cervical osteoarthritis with severe limitation of spinal mobility who showed primary open-angle glaucoma of the right eye. He had limited conversion of his head during surgery because of complications. Therefore, we performed transluminal trabeculotomy using an ophthalmic endoscope. Finally, ab interno trabeculotomy of 200 degrees was achieved by this method, and an average reduction in ocular pressure of 60% from baseline was achieved after surgery, with no major complications.

**Conclusions:**

This surgical technique may be useful as an alternative method for normal gonioscopy-assisted transluminal trabeculectomy in difficult cases.

**Electronic supplementary material:**

The online version of this article (https://doi.org/10.1186/s13256-019-2186-5) contains supplementary material, which is available to authorized users.

## Background

Gonioscopy-assisted transluminal trabeculectomy (GATT) is a new technique for ab interno trabeculotomy that was developed by Grover *et al.* [1]. GATT is sutureless and conjunctiva-sparing. Therefore, GATT is performed for treating primary congenital glaucoma (PCG) and juvenile open-angle glaucoma (JOAG) [2]. GATT is accompanied by monitoring of Schlemm’s canal using gonioscopy during surgery. To enable visibility of the surgical field, the corneal condition, critical adjustments of the patient’s head position, and the angle of the surgical microscope must be determined during the operation [1–3]. Therefore, GATT is difficult to perform in cases with limited visibility, such as with corneal opacity, sequelae of cerebral infarction, or cervical osteoarthritis. Schlemm’s canal is not able to be observed with a gonioscope in cases with severe corneal opacity. Furthermore, in cases of sequelae of cerebral infarction or cervical osteoarthritis with severe limitation of spinal mobility, changing the head position during surgery is difficult because the range of motion of the head is limited. Therefore, observing Schlemm’s canal with a conventional gonioscope is difficult in these cases. If surgeons want to perform GATT in such cases, another observation method needs to be selected to replace the gonioscope.

Surgeons often use small-gauge ophthalmic endoscopy corresponding to microincision vitrectomy surgery. In this report, we introduce a novel ab interno trabeculotomy technique in which an ophthalmic endoscope is used to compensate for the limitations of normal GATT.

## Case presentation

Our patient was a 65-year-old Japanese man who showed primary open-angle glaucoma of the right eye. Although four ophthalmic solutions (latanoprost, timolol, brinzolamide, and brimonidine tartrate) were used before surgery, intraocular pressure of the right eye was 30 mmHg, and the patient was indicated for surgery. Furthermore, because this patient had cervical osteoarthritis with severe limitation of spinal mobility, we thought that changing his head position during surgery would be difficult and performed this new surgical procedure. The patient had previously undergone cataract surgery, and an intraocular lens had been implanted. He had no particular family history or a history of allergies. His medical history included glaucoma, cervical osteoarthritis, and hyperlipidemia. Preoperative oral medications included nonsteroidal anti-inflammatory drugs for cervical osteoarthritis and atorvastatin for hyperlipidemia. He had no abnormalities in preoperative blood tests (biochemical examination and complete blood count). A physical examination showed the following: temperature, 36.2 °C; pulse rate, 76 beats per minute; respiratory rate, 22/minute; and blood pressure, 130/82 mmHg. The patient has no smoking history and drinks socially.

**Fig. 1:**
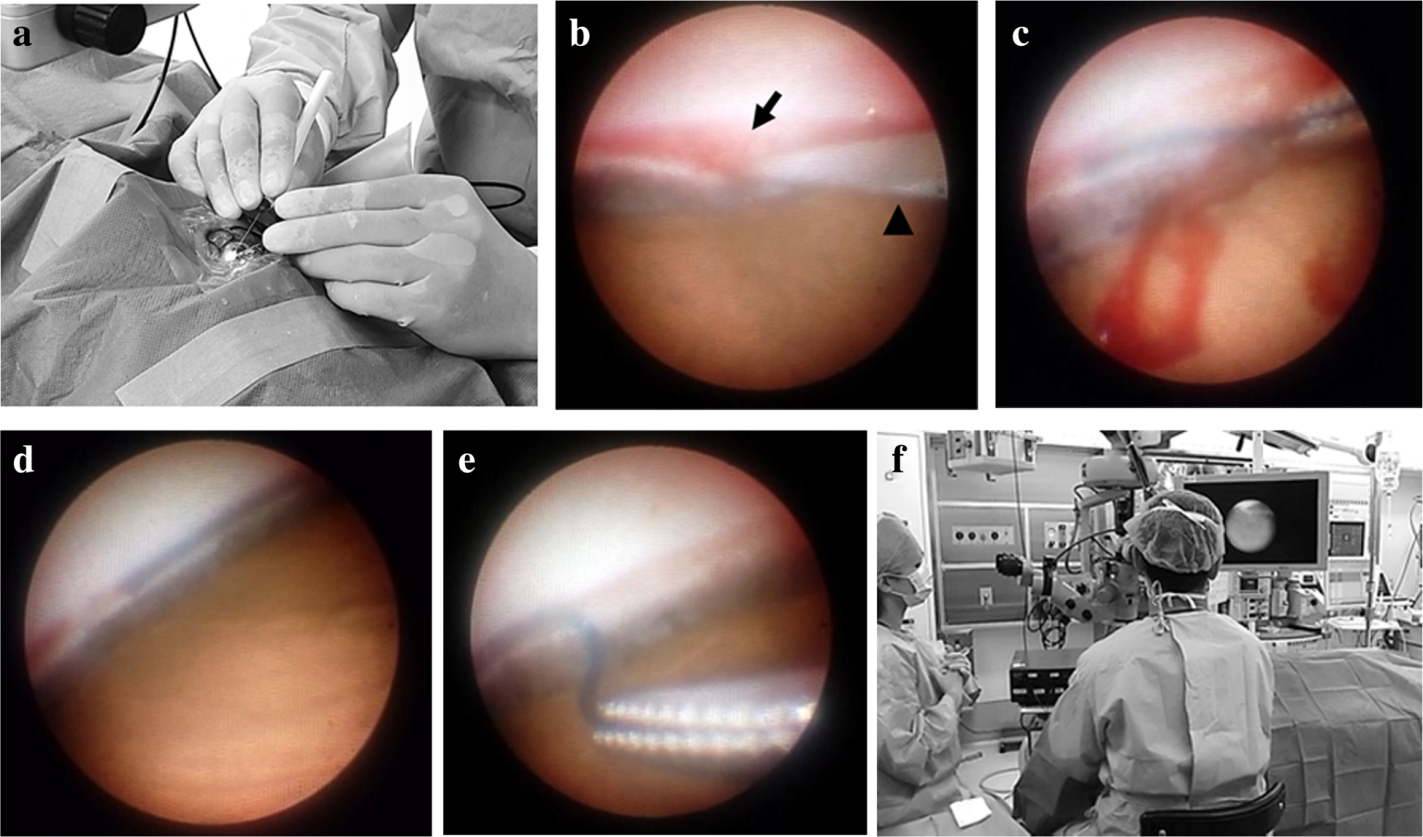
Intraoperative images of endoscope-assisted transluminal trabeculotomy. **a** Surgery was performed on the patient’s temporal side. **b** A microsurgical goniotomy incision was made using a vitreoretinal blade under endoscopic imaging guidance (*arrow*, Schlemm’s canal; *arrowhead*, microsurgical blade). The blade was inserted at the 3 o’clock position of the right eye. **c** The suture was inserted into Schlemm’s canal using microforceps. Negligible bleeding was observed during insertion of the suture. The following operation was performed by inserting the suture counterclockwise from the position of 3 o’clock of the right eye. **d** The insertion point of the suture into Schlemm’s canal on the circumference side was confirmed on the basis of changes in the location of the endoscope tip. **e** The trabeculotomy incision was made by pulling the suture while using blood that had refluxed from the goniotomy incision slot as a fulcrum. **f** The surgeon performed most of the surgery with the patient in a head-up position and used a monitor for observation

The procedure for the operation performed in our patient was as follows. The temporal side of the patient was used for the surgical approach because it was expected to allow good operability during surgery (Fig. 1a). After sterilization using standard protocols, sub-Tenon anesthesia was induced, and two corneal side ports were created. Anterior chamber stability was ensured with an ophthalmic viscosurgical device (OVD). A 23-gauge endoscope tip was then inserted from one side of the wound, and a microsurgical goniotomy incision was created with a 20-gauge vitreoretinal blade from the other side under endoscopic imaging guidance (Fig. 1b). We used an ophthalmic endoscope (FT-230F; Fiber Tech Co., Ltd., Tokyo, Japan) for this operation. The suture was inserted into Schlemm’s canal with microforceps that were used in the microsurgical goniotomy incision procedure (Fig. 1c, d). After insertion of the suture, which caused resistance, a trabeculotomy incision was made using the goniotomy incision point as a fulcrum (Fig. 1e). Normally, we operate counterclockwise and then perform the same procedure clockwise. All procedures were performed under 23-gauge endoscopic imaging guidance. Finally, irrigation and aspiration were performed to remove hyphema, including blood reflux, and an OVD in the anterior chamber. At the end of the surgery, there was no need to suture the side ports of the cornea. The patient’s head position and the angle of the surgical microscope were not adjusted during surgery. Use of a surgical microscope is essential for viewing the entire surgical field, creating corneal side ports, and performing irrigation and aspiration of the anterior chamber. The surgeon performed most of the surgery with the patient in a head-up position, using the monitor for observation (Fig. 1f). Finally, ab interno trabeculotomy of 200 degrees was achieved by this method.

The preoperative intraocular pressure was 30 mmHg, but it was reduced to 12 mmHg the day after surgery. Mild hyphema was confirmed the day after surgery, but it was improved with only observation. There was no change in corneal endothelial cell density from before to after surgery. Twenty months after surgery, the intraocular pressure was controlled at 15 mmHg with the use of one ophthalmic solution (latanoprost).

## Discussion and conclusions

In this report, we describe the ab interno trabeculotomy technique using an ophthalmic endoscope for a patient with glaucoma with cervical osteoarthritis and severe limitation of spinal mobility. This condition made changing the head position difficult, and normal GATT could not be performed.

GATT is a minimally invasive surgical procedure involving conjunctiva-conserving surgery [1–3]. In particular, GATT shows apparent advantages of surgery for PCG and JOAG operations because management after surgery is easier [3]. GATT should be performed under a surgical microscope using gonioscopy. Therefore, GATT is difficult to perform using gonioscopy in patients with corneal opacities or problems with intraoperative head positioning, such as sequelae of cerebral infarction and cervical osteoarthritis with severe limitation of spinal mobility. Consequently, the greatest advantage of our new technique is that transluminal trabeculotomy can be performed in cases with corneal opacities and in cases where intraoperative head repositioning is not possible. This surgical technique is a modified version of GATT, in which observation is optimized using an ophthalmic endoscope during surgery. Another advantage of using an endoscope is that checking the insertion range of the suture is easy.

However, there are several problems with this technique. One disadvantage is that intraoperative visibility with an endoscope is inferior to observation with gonioscopy. Another disadvantage is the observation range of Schlemm’s canal with an endoscope. In this procedure, there is an unobservable viewing angle on the side of endoscope insertion. Therefore, when making an incision at an angle > 250 degrees, a new endoscope insertion site must be created. By changing the position of the corneal side port and the angle of the endoscope, a considerable range of Schlemm’s canal can be observed, regardless of the patient’s condition.

An additional limitation of this surgical technique is a reduction in intraoperative visibility due to bleeding when a microsurgical goniotomy incision is created before insertion of the suture, as in GATT. If intraoperative visibility is insufficient, there is a risk that incorrect insertion of the suture may occur. If a small amount of bleeding at the time of incision makes inserting the suture difficult, a small amount of an OVD can be inserted at the incision site to ensure visibility. Therefore, when heavy bleeding reduces intraoperative visibility, it should be mitigated by irrigation and aspiration in the anterior chamber, as well as by subsequent maintenance of anterior chamber stability with an OVD. If controlling the bleeding that has occurred during surgery is difficult, a decision needs to be made not to continue the surgery forcibly, but to stop the operation instead.

Furthermore, if there is resistance after insertion of the suture into Schlemm’s canal, there is a risk of misinsertion if it is inserted forcibly. Therefore, in such a case, a decision needs to be made regarding switching to suture insertion from the reverse side to achieve an incision of at least 120 degrees in total (Additional files 1 and 2). If resistance at the time of insertion suddenly decreases and insertion becomes easy, misinsertion should be suspected, and the tip of the suture should be confirmed with an endoscope.


**Additional file 1:** Intraoperative video of our patient’s case. (WMV 17653 kb)



**Additional file 2:** Video of an additional case. The video shows a 76-year-old man with exfoliation glaucoma of the right eye who had experienced a stroke and could not easily adjust his head position. In this patient, ab interno trabeculotomy of 220 degrees was achieved with endoscope-assisted transluminal trabeculotomy, and an average reduction in ocular pressure of 67% from baseline was achieved after surgery with no complications. This was the first eye surgery for this patient. (WMV 22414 kb)


Numerous surgical procedures using an ophthalmic endoscope have been reported for glaucoma surgery [4–8]. Using an ophthalmic endoscope is effective for observation during surgeries, including goniotomy for PCG [5], goniosynechialysis for synechial angle closure glaucoma [6, 7], and viscocanalostomy [8]. However, observations made using an ophthalmic endoscope are inferior to those made with a surgical microscope in terms of definition and solidity. Therefore, considerable experience is necessary when using and handling an ophthalmic endoscope freely during surgery. We used a 23-gauge ophthalmic endoscope for our patient. However, further minimally invasive surgery may also be possible by using a 25- or 27-gauge ophthalmic endoscope.

In conclusion, our new surgical technique is a useful method for treating cases with difficult or limited visibility during GATT.

## Abbreviations

GATT: Gonioscopy-assisted transluminal trabeculectomy
JOAG: Juvenile open-angle glaucoma
OVD: Ophthalmic viscosurgical device
PCG: Primary congenital glaucoma
